# Complete resolution of recalcitrant periungual verruca vulgaris using laser-assisted drug delivery

**DOI:** 10.1016/j.jdcr.2025.09.018

**Published:** 2025-09-26

**Authors:** Aneri Bhargav Patel, Andrew Creadore, Katerina Yale, Christopher B. Zachary

**Affiliations:** a Department of Dermatology, University of California, Irvine, California; b University of California, Davis, School of Medicine, Sacramento, California

**Keywords:** cidofovir, digital warts, Er:YAG, laser, laser-assisted drug delivery, recalcitrant verruca vulgaris, topical cidofovir, verruca vulgaris, warts

## Introduction

Verruca vulgaris (VV), also known as the common wart, is linked to human papillomavirus (HPV) genotypes 2 and 4. VV is benign and affects up to 30% of children and 12.9% of adults, with disease duration potentially lasting 2 years or more.^1^ There are various guidelines regarding treatment approaches for VV; most commonly, first-line treatments include salicylic acid, cryotherapy, topical 5-fluorouracil, cantharidin, podophyllin solution, and duct tape occlusion.^2^ Cidofovir, an antiviral agent that induces apoptosis of HPV-infected cells, is a topical treatment that has shown promise in the setting of VV.^3^ Prior studies have shown successful treatment of recalcitrant VV with intralesional cidofovir; however, this approach often requires multiple painful injections. VV in the periungual locations has a hyperkeratotic surface that limits the efficacy of topical treatments due to poor drug penetration. In such cases, the use of an ablative laser can facilitate drug delivery by disrupting the stratum corneum and allowing the topical agent to reach the base of the lesion more effectively.

Herein, we present a patient with recalcitrant VV successfully treated with laser-assisted drug delivery (LADD) using erbium:yttrium-aluminum-garnet (Er:YAG) laser therapy and topical cidofovir, achieving 100% clearance of the lesion. Although LADD with topical cidofovir has been described in the treatment of VV, this is an uncommon case demonstrating successful treatment of a periungual wart using this method.^4^

## Case report

**Fig 1:**
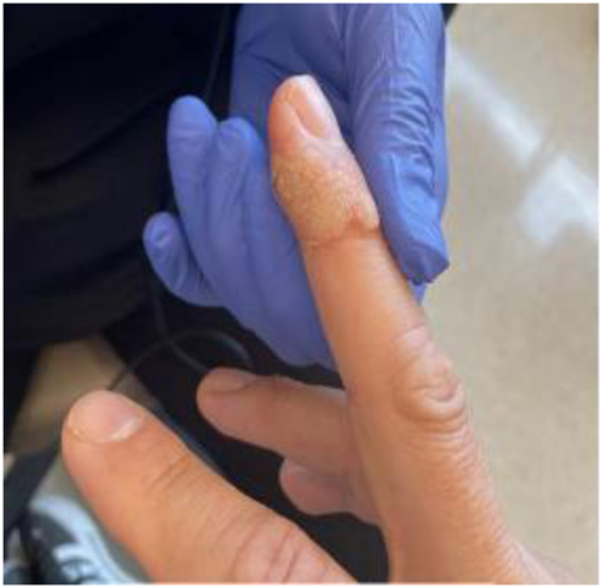
Prior to erbium:yttrium-aluminum-garnet treatment, the right middle finger has rough, irregular, firm yellow-to-brown nodules present on the area of the middle and distal phalanx.

A 49-year-old male patient with Fitzpatrick III skin type and no significant medical history presented with a 2-year history of a 1.5-cm verruca vulgaris on the right middle finger. Past treatments included several rounds of cryotherapy, intralesional candida injections, and multiple topical therapies, including imiquimod 5% cream, 5-fluorouracil 5% cream, tazarotene 0.1% gel, cantharidin, and compounded high-dose salicylic acid, podophyllin, and pulse dye laser, all of which had failed to improve the lesion (Fig 1). Given the lack of improvement with multiple treatments and a recalcitrant lesion, the option of LADD, namely fractionated Er:YAG, ablative laser, followed by topical cidofovir 3% ointment was discussed, to which the patient agreed.

Treatment was performed with a 2940-nm Er:YAG fractionated ablative laser with a depth of 450 μm, density of 5.5%, and scan size 1 to 2 mm. Of note, no topical or local anesthetic was required prior to laser ablation. Topical cidofovir ointment was applied immediately posttreatment, and he was instructed to continue with this twice daily under occlusion.

**Fig 2:**
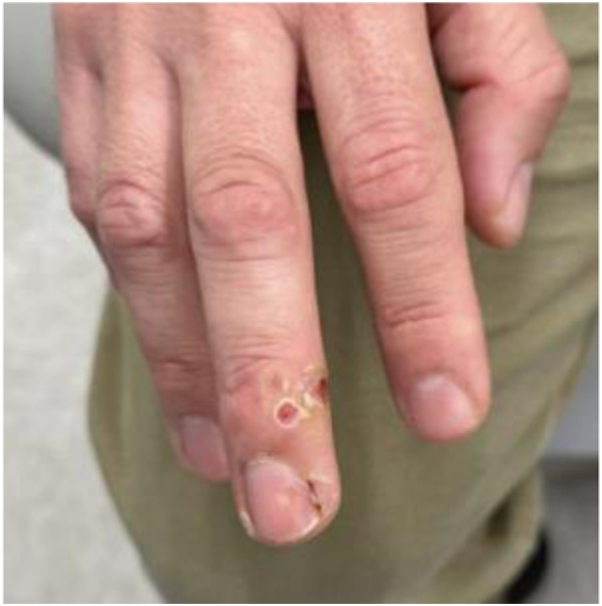
Right middle finger with mild ulceration, reddening, and some hyperkeratosis of areas of previous verruca vulgaris. These photographs were taken after 3 treatments.

At his follow-up visit 2 weeks later, he received another treatment with fractionated Er:YAG with the same settings and topical cidofovir. He continued using topical cidofovir twice daily at home. After another 3 weeks, he received a third round of Er:YAG with the same parameters. At this time, he noted near resolution of the VV. At his last (fourth) treatment 2 weeks later, he had another round of fractionated Er:YAG at a depth of 450 μm and 5.5% density (Fig 2).

**Fig 3:**
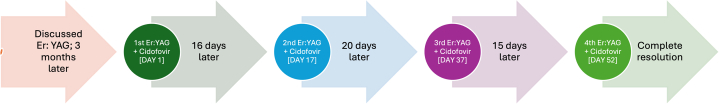
Treatment timeline for the case. *Er:YAG*, Erbium:yttrium-aluminum-garnet.

**Fig 4:**
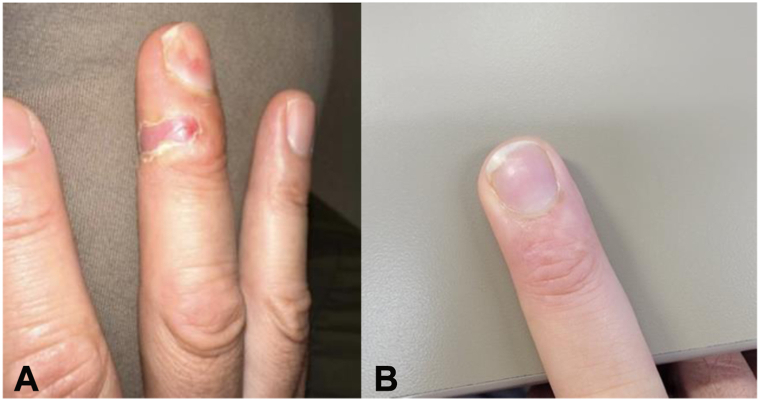
**A,** Periungual lesion on the dorsal aspect of the long finger. The lesion appears as a well-demarcated and slightly erythematous. Surrounding the lesion is desquamation indicative of re-epithelialization, consistent with newly formed epidermis during the healing process. **B,** Well-healed treatment site, no evidence of verruca vulgaris.

He was seen for his last follow-up appointment about 2 weeks after 4 treatments with cidofovir by LADD (Fig 3). On examination, on the right long finger, he had a 1-cm erosion with desquamation on the edges, with no evidence of residual wart along with some onychodystrophy along lateral aspect of the right middle fingernail (Fig 4, *A*). Note that he had complete resolution of VV and excellent healing of the skin surrounding the area (Fig 4, *B*).

## Discussion

A multitude of treatments and guidelines have been established for VV; however, some cases of recalcitrant VV do not respond to traditional treatment modalities and can significantly affect a patient’s quality of life.^2^ A prior study demonstrated that topical cidofovir can have a 55.2% clearance rate when used alone, with no recurrence of lesions observed between 2 months and 3 years’ posttreatment. This highlights its role as an effective treatment option for recalcitrant nongenital VV.^4^^,^^5^ Similarly, studies have explored the use of the Er:YAG laser for male genital warts, in which patients undergoing multiple sessions for genital warts achieved good success rates; 61 out of 102 patients achieved a complete response in 12 months of treatment.^6^

LADD has a broad range of applications, and a recent systematic review demonstrated its efficacy in enhancing the topical absorption of drugs. Fractional lasers (ablative and nonablative) are commonly utilized in combination with topical agents for LADD.^7^ The review also mentions how the side effects seen from LADD are oftentimes the side effects from the laser itself, including pain, crusting, and erythema. Of note, to our knowledge, none of the studies reported any systemic side effects or systemic adverse events, including no systemic toxicity from the absorption of the topicals.^7^

Coates et al^4^ reported 2 cases of plantar VV treated with Er:YAG and topical cidofovir. In the first case, there was a 60% reduction in tumor size with areas of complete clearance after 9 treatments with Er:YAG and cidofovir, whereas the second case underwent 4 treatments of pulse dye laser with no improvement followed by 5 sessions of Er:YAG and cidofovir, showing 80% clearance on the sole of the right foot, with the fourth toe on the right foot achieving 100% clearance. The use of Er:YAG LADD of cidofovir for periungual VV has not been well documented; our case demonstrates successful treatment with 100% resolution, highlighting an uncommon and effective approach for a challenging lesion location.

The mechanism behind the success of LADD is likely related to the many microchannels with diameters of 430 μm and depth of 450 μm created by the laser which facilitate the penetration of topical cidofovir, allowing the drug to reach the entire viral wart more effectively.^4^^,^^8^^,^^9^ An additional advantage of this approach was the excellent tolerability; the patient reported minimal discomfort during the procedure, did not require topical or local anesthesia, and required only minimal wound care, allowing him to continue his daily activities without interruption between sessions.

This case demonstrates complete resolution of recalcitrant periungual VV following treatment with LADD using fractionated Er:YAG laser and topical cidofovir. The excellent clinical outcome, coupled with minimal discomfort and downtime, underscores the potential of this modality as an option for treatment-resistant warts. Further studies are warranted to validate its efficacy in broader patient populations.

## Conflicts of interest

None disclosed.
